# Olive Cake Powder as Functional Ingredient to Improve the Quality of Gluten-Free Breadsticks

**DOI:** 10.3390/foods11040552

**Published:** 2022-02-15

**Authors:** Giuditta de Gennaro, Graziana Difonzo, Carmine Summo, Antonella Pasqualone, Francesco Caponio

**Affiliations:** Department of Soil, Plant and Food Science (DISSPA), University of Bari Aldo Moro, Via Amendola, 165/a, I-70126 Bari, Italy; giuditta.degennaro@uniba.it (G.d.G.); carmine.summo@uniba.it (C.S.); antonella.pasqualone@uniba.it (A.P.); francesco.caponio@uniba.it (F.C.)

**Keywords:** gluten-free, breadsticks, olive cake, dietary fibre, polyphenols, antioxidant activity, lipid oxidation, texture

## Abstract

The growing demand for high-quality gluten-free baked snacks has led researchers to test innovative ingredients. The aim of this work was to assess the feasibility of olive cake powder (OCP) to be used as a functional ingredient in gluten-free (GF) breadsticks. OCP was used by replacing 1, 2, and 3% of maize flour into GF breadstick production (BS1, BS2, BS3, respectively), and their influence on nutritional, bioactive, textural, and sensorial properties was assessed and compared with a control sample (BSC). BS1, BS2, and BS3 showed a higher lipid, moisture, and ash content. BS2 and BS3 had a total dietary fibre higher than 3 g 100 g^−1^, achieving the nutritional requirement for it to be labelled as a “source of fibre”. The increasing replacement of olive cake in the formulation resulted in progressively higher total phenol content and antioxidant activity for fortified GF breadsticks. The L* and b* values decreased in all enriched GF breadsticks when compared with the control, while hardness was the lowest in BS3. The volatile profile highlighted a significant reduction in aldehydes, markers of lipid oxidation, and Maillard products (Strecker aldehydes, pyrazines, furans, ketones) in BS1, BS2, and BS3 when compared with BSC. The sensory profile showed a strong influence of OCP addition on GF breadsticks for almost all the parameters considered, with a higher overall pleasantness score for BS2 and BS3.

## 1. Introduction

The gluten-free (GF) market is growing rapidly due to an indistinct adherence of consumers to a gluten-free diet [1]. Adherence to a GF diet is not limited to people who are intolerant to gluten, but is also observed by people who are not affected by gluten disorders who decide to follow it, believing that GF products are healthier, or simply following a trend of the moment [2]. However, several studies have focused attention onto the nutritional imbalance of GF cereal products [3,4,5,6]. From the comparison with their gluten-containing counterparts, GF products are richer in fats, mainly saturated, and carbohydrates, with a higher glycaemic index [5,7,8], and they are poorer in protein, dietary fibre, minerals, and vitamins [3,4,5,9,10].

The absence of gluten, beyond just negatively affecting the nutritional profile, has a strong influence on even the rheological, textural, and sensory properties, determining a relaxed dough that is difficult to handle, and a consequently crumbling texture, a poor colour, and a low satisfying taste and mouthfeel of baked products [11]. Indeed, gluten plays a fundamental role in formulating high-quality cereal-based goods: it is formed of proteins, mainly glutenin and gliadin, so that when fully hydrated they confer cohesive elastic and viscous properties to the dough, positively affecting the textural and sensory properties of baked foods [12,13].

GF cereal food deficiencies have led researchers to find alternative ingredients to improve their quality. Several ingredients have been tested, including starches [14,15], hydrocolloids [16,17], isolated protein or protein-rich ingredients [18,19,20,21], and dietary fibre, e.g., inulin, psyllium fibre, and resistant starch [15,22]. Among them, protein and dietary fibre addition allows us to meet the need for the nutritional and technological improvement, due to their functional properties [23,24,25]. However, alternatively to the use of the functional ingredients mentioned above, which have a high cost [26], vegetable by-products could represent a valuable and sustainable source of bioactive and functional compounds with technological properties [27,28,29]. Olive by-products seem to be very promising [30,31], and among them, olive cake obtained by a multi-phase decanter and constituted only by pulp and its vegetation water without traces of kernels [32], is an interesting source of dietary fibre, polyphenols, triterpenic acids, tocochromanols, and carotenoids [33,34,35]. Moreover, it shows a good mineral content and lipidic profile due to its richness in mono and polyunsaturated acids (oleic, linoleic, and linolenic acid) [36]. In the literature, there are several studies that have highlighted the health benefits related to a regular and appropriate ingestion of fibre [37,38,39,40] and phenolic compounds [41,42,43], which are also exploited as natural additives in foods, substituting chemical ones, due to their antioxidant and antimicrobials actions [44,45,46].

Some studies have been conducted on the exploitation of olive cake as a new functional ingredient in fortified cereal-based foods [31,47,48], but no research has been carried out using olive cake powder as a new ingredient in gluten-free products. In this framework, the aim of this study was to use olive cake powder in the formulation of gluten-free breadsticks to improve their quality. In particular, olive cake was added at 1, 2, and 3% in substitution of maize flour into GF breadstick production, and the influence on the textural, sensory, and nutritional properties was evaluated. 

## 2. Materials and Methods

### 2.1. Gluten-Free Breadsticks Preparation

Control GF breadsticks (BSC) were prepared using the following ingredients: rice flour (41%), water (35%), sunflower oil (11%), maize flour (9%), psyllium fibre (2%), baking powder (1.5%; disodium diphosphate, sodium hydrogen carbonate, maize starch), and salt (0.5%). Olive cake powder (OCP)—prepared by lyophilizing (Lyophiliser Buchi, Switzerland, Lyovapor^TM^ L-200) wet olive cake and subsequently grinding it—was utilised by replacing maize flour, which mainly affects the sensorial properties, in amounts of 1, 2, and 3% (BS1, BS2, and BS3, respectively). For the breadsticks’ preparation, psyllium fibre was gradually added to water and oil and homogenised to facilitate the formation of a gel. The resulting gel was added to rice and maize flours, OCP, salt, and baking powder previously mixed in a bowl. After 15 min of manual kneading, the doughs were hand-shaped to form breadsticks 9 cm long. Afterwards, the breadsticks were boiled in water for 2 min and let to dry for 5 min at room temperature to determine the starch gelatinisation and to obtain a more compact and cohesive structure of the breadsticks. Finally, the boiled breadsticks were baked in a forced-air convention oven (Smeg SI 850 RA-5 oven, Smeg S.p.A., Guastalla, Italy) at 200 °C for 27 min and cooled to room temperature. Three independent breadsticks’ productions were carried out.

### 2.2. Proximate Composition

Protein (total nitrogen × 6.25), ash, and lipids content were determined using the AOAC methods 979.09, 923.03, and 945.38 F, respectively [49]. Moisture was determined by a moisture analyser (Mod. MAC 110/NP, Radwag Wagi Elektroniczne, Poland) at 105 °C, until a constant weight. The total fibre content was determined by an enzymatic–gravimetric process as described by the AOAC Official Method 991.43 [49]. The carbohydrate content was calculated as a difference. The analyses were carried out in triplicate. 

### 2.3. Extract Preparation

The phenolic extraction was carried out following the procedure reported by Caponio et al. [50] with some modifications. An amount of 2 mL of hexane, to remove the oil phase, was added to 2 g of sample and 10 mL of methanol:water (80:20 *v*/*v*). After stirring for 15 min, samples were sonicated (Ultrasonic cleaner CP104, EIA) for 15 min, then centrifugated at 10,000× *g* min^−1^ for 10 min at 24 °C (Thermo Fisher Scientific, Osterodeam Harz, Germany). The supernatant was collected, while the pellet was subjected to two further extractions. The extracts were centrifugated at 8000× *g* min^−1^ for 2 min at 24 °C. Then, the hydroalcoholic phases were filtered through a nylon filter (pore diameter 0.45 μm, Sigma, Ireland) within amber vial. A total of three extractions were carried out, following the procedure described above. 

#### 2.3.1. Total Phenols and Antioxidant Activity of OCP and GF Breadsticks

Total phenols content and antioxidant activity were determined spectrophotometrically, by the Folin-Ciocalteu method and ABTS (2, 2′-azinobis-3-ethylbenzothiazoline-6-sulfonic acid) and DPPH (1,1-diphenyl-2-picrylhydrazyl)assays, respectively. An amount of 20 μL of extract, properly diluted, was added to 980 μL of MilliQ water and 100 μL of Folin reagent. After 3 min of incubation, 800 μL of Na_2_CO_3_ was added and kept in the dark for 1 h. The absorbance was read at 720 nm using a Cary 60 spectrophotometer (Cernusco, Milan, Italy). The results were expressed as milligrams of gallic acid equivalents (GAE) per gram of product. As regards antioxidant activity, the DPPH (2,2-diphenyl-1-picrylhydrazyl) and ABTS assays were performed as reported by Difonzo et al. [51]. The results were expressed as micromolar Trolox equivalent (TE) g^−1^ dry weight. The determinations were carried out in triplicate. 

#### 2.3.2. Total Tocopherols of OCP

Tocopherols were determined using a reverse-phase, ultra-high-performance chromatography-fluorescence detector (RP-UHPLC-FLD). An amount of 0.02 g of sample was dissolved in 700 µL of 2-propanol, then stirred and filtered. Then, 20 µL was injected into a UHPLC system (Dionex Ultimate 3000 RSLC, Waltham, MA, USA) equipped with an HPG-3200 RS pump, WPS-3000 autosampler, TCC-3000 column compartment, and a FLD-3400 RS fluorescence detector. A Dionex Acclaim 120 C18 analytical column (150 × 3 mm i.d.) with a particle size of 3 µm (Thermo Scientific, Waltham, MA, USA) was used. Mobile phase consisted of a mixture of acetonitrile and methanol (1:1 *v*/*v*) at a constant flow rate of 1 mL·min^−1^ in isocratic elution mode. The FLD detector was set at an excitation wavelength of 295 nm and at an emission wavelength of 325 nm. The quantification of tocopherols was performed by external calibration obtained with standard solutions of α-tocopherols, and the results were expressed as mg·kg^−1^ of sample. 

### 2.4. Texture Analysis of GF Breadsticks

The texture parameters (hardness and brittleness) of GF breadsticks were determined through a three-point bending test according to Conte et al. [52], providing some modifications. A texture analyser, Z1.0 TN (Zwick/Roell, Ulm, Germany), was equipped with a 1 KN load cell. The breadsticks were placed in the middle of the support, setting the bars distance at 60 mm and the blade probe speed at 3 mm s^−1^. The maximum force required to break the breadstick (N) and the distance crossed by the blade before breadsticks broke (mm) were measured, which represent hardness and brittleness, respectively. The analysis was carried out in triplicate. 

### 2.5. Colour Evaluation

The colorimetric analysis was carried out using a colorimeter CM-600d (Konica Minolta, Tokyo, Japan) supported by SpectraMagic NX software. Gluten-free breadsticks were ground to obtain a powder and placed in a specific vessel. Luminosity (L*), red index (a*), and yellow index (b*) were considered in accordance with the International Commission on Illumination (CIE). Three replicated analyses were carried out. Colour difference (ΔE) between control (*c*) and fortified samples was calculated as follows:

(1) $$\Delta E={\left[(L-Lc{)}^{2}+{\left(a-ac\right)}^{2}+{\left(b-bc\right)}^{2}\right]}^{1/2}$$

### 2.6. Volatile Compounds of GF Breadsticks

Volatile compounds of GF breadsticks were analysed through headspace solid phase micro-extraction (HS-SPME), coupled with a gas chromatography/mass spectrometry as reported by Difonzo et al. [53]. In particular, 0.5 g grounded GF breadsticks were weighed in 12 mL vials and 150 μL of 1-propanol was added as internal standard plus 4 mL of NaCl (20% *w*/*v*) aqueous solution. Vials were sealed with aluminium crimps caps equipped with a butyl rubber septum. Before volatile compounds’ extraction, vials were shaken for 2 min with a laboratory vortex to promote samples homogenisation. For the separation of volatile compounds, an Agilent 6850 gas-chromatograph equipped with an Agilent 5975 mass-spectrometer (Agilent Technologies Inc., Santa Clara, CA, USA) and an HP-Innowax (Agilent Technologies Inc., Santa Clara, CA, USA) polar capillary column (60 m length × 0.25 mm i.d. × 0.25 μm film thickness) was used. Volatiles were extracted by exposing a SPME fibre 75 μm carboxen/polydimethylsiloxane (CAR/PDMS) (Supelco, Bellefonte, PA, USA) in the headspace of the vials at 40 °C for 50 min, then it was desorbed for 6 min in the injection port of the gas-chromatograph, operating in a split-less mode at 230 °C for 3.5 min. The separation of volatile compounds was carried out following the current conditions: injector temperature, 250 °C; helium was used as a carrier gas with a flow of 1.5 mL min^−1^. The oven temperature was held for 5 min at 35 °C, then increased by 5 °C min^−1^ until it reached 50 °C. This temperature was held constant for 5 min, then raised to 210 °C at 5.5 °C min^−1^, and finally held constant at 210 °C for 5 min. The mass detector was set at the following conditions: interface temperature 230 °C, source temperature 230 °C, ionisation energy 70 eV, and scan range 33–260 amu. The volatile compounds were quantified through the standardisation of the peak area of internal standard with the peak areas of the compounds of interests. The analyses were carried out in triplicate. 

### 2.7. Sensory Evaluation

Sensory analysis was conducted by a semi-trained panel composed of eight panellists, chosen among professors, laboratory technicians, and researchers. The analysis was carried out according to the ethical guidelines of the Laboratory of Food Science and Technology of the Department of Plant, Soil and Food Science of University of Bari, Italy. A total of 10 descriptors were selected considering the product analysed and by-products added in the formulation: 3 descriptors for visual appearance (surface uniformity, surface colour, surface colour intensity); 1 descriptor for the odour (olive); 2 for texture perceived during tasting (hardness, brittleness); 4 for taste (salty, bitterness, astringent, oiliness). The perception intensity of the descriptors was assessed on a scale ranging from 0 (no perception) to 9 (maximum intensity). Finally, the panellists evaluated the overall pleasantness of the breadsticks using the same scale as the descriptors analysed. 

### 2.8. Statistical Analysis

A one-way analysis of variance (ANOVA) followed by the Tukey’s HSD test were carried out using the Minitab Statistical Software (Minitab Inc., State College, PA, USA).

## 3. Results and Discussion

### 3.1. Proximate Composition of OCP and GF Breadsticks

As reported in Table 1, OCP was characterised by a moisture content of 2.32 g 100 g^−1^ and a high lipid content (mainly monounsaturated), as found by Lozano-Sánchez et al. [36]. OCP also showed a good protein content, 7.68 g 100 g^−1^, which was in line with the result obtained by Nunes et al. [54], and ash was equal to 8.97 g 100 g^−1^. 

A reasonable total dietary fibre was found (23.45 g 100 g^−1^), but it was lower than in other studies [55,56]. Moreover, OCP was also a good source of bioactive compounds [57], with values of total phenols and tocopherols equal to 78.23 mg GAE g^−1^ and 412.24 mg kg^−1^, respectively, which contribute to the high antioxidant activity encountered with DPPH and ABTS assays, equal to 285.24 and 346.12 µmol TE g^−1^, respectively. 

Regarding the GF breadsticks, the addition of OCP positively influenced their quality (Table 2). In particular, the highest content of total dietary fibre was found in BS3 (3.59 ± 0.13 g 100 g^−1^), followed by BS2 samples (3.16 ± 0.08 g 100 g^−1^), while BSC and BS1 showed lower values without significant differences. Therefore, both BS2 and BS3 can be labelled as a “source of fibre” in accordance with the EC Regulation n.1924/2006 [58], which states that a food may be labelled as a source of fibre if it contains at least 3g of fibre per 100 g or 1.5 g per 1000 kcal. The higher moisture content observed in the fortified samples could be related to the higher content of fibre known to have water holding and binding capacities [59]; indeed, the same trend was observed by other authors after the addition of fibrous ingredients [60,61,62]. Even for ash content, BS3 showed a statistically higher content (2.67 ± 0.04) than for other samples; this result could be reasonably associated with OCP addition, considering the mineral richness of olive cake [63]. Finally, protein and carbohydrate content significantly decreased in the fortified samples (BS1, BS2 and BS3) in comparison with BSC, due to the increase in the other parameters considered. 

### 3.2. Phenolic Compounds and Antioxidant Activity of GF Breadsticks

**Figure 1:**
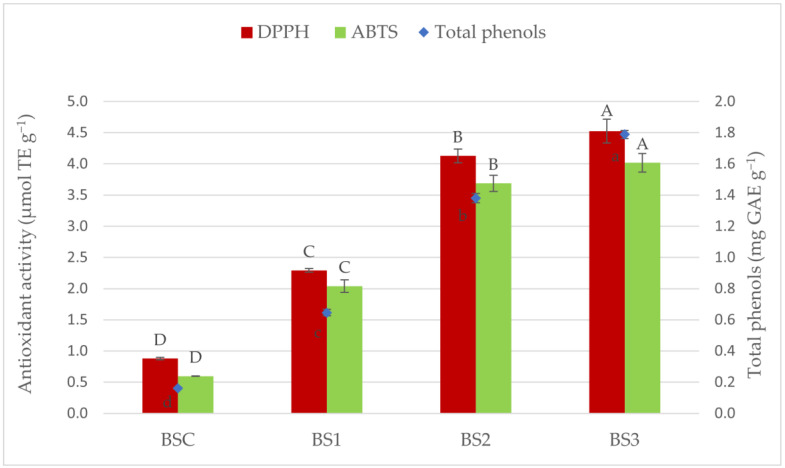
Antioxidant activity and total phenols determination in breadsticks. Different letters show significantly different means of data (*p* < 0.05) according to one-way ANOVA and the Tukey’s HSD test. Capital letters refers to antioxidant activity and lowercase letter to total phenols content. Abbreviations: BSC, control breadsticks without OCP; BS1, BS2, BS3, breadsticks with 1, 2, and 3% olive cake powder replacing maize flour, respectively.

The maize flour replacement with OCP significantly affected the total phenolic content (TPC) and antioxidant activity (AA) of baked GF breadsticks as reported in Figure 1. A dose-dependent increase in TPC and AA with a higher replacement rate of OCP in the GF breadstick formulation was observed. The significant highest value of TPC was found in the BS3 sample (1.79 ± 0.03 mg GAE g^−1^), while BSC showed the lowest one (0.16 ± 0.01 mg GAE g^−1^), and the same trend was found for antioxidant activity. The DPPH values were found in a range of 4.52 ± 0.19 µmol TE g^−1^ and 0.88 ± 0.02 µmol TE g^−1^ in BS3 and BSC, respectively. Similarly, also for ABTS, the highest value was found in the BS3 sample, which decreased progressively in the other formulations, reaching the lowest value in the control sample (0.60 ± 0.01 µmol TE g^−1^). Therefore, considering the high phenol content in OCP (75.56 ± 3.34 mg GAE g^−1^), the data obtained highlight a positive correlation between OCP addition and the increase in total phenol content and antioxidant activity found in fortified GF breadsticks. Likewise, the addition of olive cake in fish burgers, pasta, and bread also resulted in an increase in phenol content and antioxidant activities [48,56,64]. In addition to olive cake, the addition of other by-products such as apple pomace or grape pomace, as reported by Mir et al. [65] and Rainero et al. [66], respectively, into brown rice flour crackers and wheat breadsticks also enhanced their bioactive content. Pomegranate seed powder has also proven to be an excellent source of phenolic compounds; thus, the addition of increasing amounts (5–10%) into gluten-free bread entailed a higher total phenol content and antioxidant activity of fortified bread than the control. Similarly, blackcurrant residue added into gluten-free chocolate cookies revealed an interesting potential for enhancing their bioactive content, and furthermore, the authors determined that a large amount of compounds with antioxidant activity could reach the large intestine intact, exerting their antioxidant function until excretion [60].

### 3.3. Colorimetric and Textural Properties of GF Breadsticks

The colour and textural properties are fundamental properties for foods’ acceptability, particularly for GF products such as bread, pasta, and snacks. These products are usually characterised by a crumbling structure and pale colours [23]. The colour of GF breadsticks was significantly influenced by the addition of OCP (Table 3). Specifically, L* and b* decreased significantly with increasing OCP addition. The browning of GF breadsticks is strictly related to the colour of OCP (brown) added in the formulations. Similar results were obtained by Jahanbakhshi et al. [67] with the addition of olive stone powder into sponge cake, which caused a reduction in L* and b* parameters. Likewise, the colour parameters of gluten-free snacks based on brown rice and amaranth flour added with cactus pear peel powder produced by Miranda et al. [68] were also influenced by the addition of the by-product. Specifically, a reduction in luminosity (L*), but an increase of parameter a*, were observed, indicating a shift towards red shades with an increasing amount of cactus pear powder addition. These results emphasise how the raw material colour plays a key role in influencing the colour of finished products. Finally, the total colour difference (ΔE) highlighted the colour difference between the fortified (BS1, BS2, BS3) and control samples (rice and maize flour), indicating a growing difference with an increasing percentage of olive cake added. In addition to OCP addition, Maillard reactions, which occur in high-temperature-treated products, contribute to the colour, flavour, aroma, and texture of the end product [69]. 

Maillard reactions take place between amine groups, in amino acids and proteins, and carbonyl compounds, which usually come from the reducing of sugars (fructose, glucose, maltose, or lactose) [70]. The colour development is ascribed to the final stage of the Maillard reaction, where the condensation of carbonyls and amines provokes the formation of brown-coloured, high-molecular-weight compounds, named melanoidins [71,72].

Hardness and brittleness are common parameters considered for the evaluation of the textural properties of baked snacks, being closely related with their freshness and wholesomeness [73]. As reported in Table 3, BS3 showed the lowest value of hardness (12.68 N) when compared with BS2, BS1, and BSC that all showed no significant differences. Among the factors which can affect the textural parameters of bakery foods, lipids exert a fundamental role, acting as a lubricant. However, their impact on GF bakery foods is less relevant for the absence of interactions with the gluten network [74]. 

According to the literature, flour particle size may have affected the structure of the final product; specifically, the addition of coarse flours led to a hardness reduction of several bakery products [75,76,77,78]. From the results obtained by Mancebo et al. [78], it was observed that gluten-free cookies prepared with coarse-grained flour required a significant lower peak force than cookies prepared with fine-grained flours. A similar result was obtained by Belorio et al. [76], who assessed that hardness was most affected by particle size, followed by diameter and spread factor. Specifically, the authors observed a reduction in hardness with an increasing percentage of coarse maize flour inclusion in the preparation of gluten-free cookies. Therefore, the addition of a large amount of coarse and uneven OCP could have determined the formation of a more fragile structure, which positively affected hardness by reducing it. Moreover, the textural properties of final products may be affected by the nature of the fibres (soluble or insoluble), as well as size, morphology, and technological properties such as water binding and holding capacity, which can affect the water content of the end product [62,79,80]. According to Šarić et al. [81], the positive correlation existing between a high amount of water in the system and the lower breaking force (hardness) of gluten-free cookies with added blueberry pomace derives from the penetration of water in the starch granules, which swelled. This phenomenon determined an increase in intramolecular bond breakage and macromolecule mobility, with a consequently less-dense cookie structure and lower breaking force. On the contrary, a reduction in volume and firmer crumbs occurred in gluten-free bread to which quinoa bran was added [82]. According to the authors, a possible explanation is the competition of dietary fibre and starch for water absorption and the resulting limited starch swelling and gelatinisation. Similarly, Raczyk et al. [83] observed an increase in bread hardness after 48 and 72 h of storage with coconut and chestnut flour supplementation, because of fibre and sugar increases. Regarding brittleness, it refers to the distance crossed by the tool before the breadsticks breaks. For this parameter, no significant differences were found among the samples, although BS3 displayed a slightly higher brittleness as a consequence of its lower hardness value [84].

### 3.4. Volatile Profile of GF Breadsticks

For baked foods such as biscuits, crackers, and breadsticks, lipid oxidation represents one of the main causes of quality deterioration [85], and pentanal, hexanal, *trans*2-heptanal, and nonanal are aldehydes considered to be markers of lipid oxidation [86,87,88]. Table 4 reports the volatile compounds of GF breadsticks and a total of 44 volatile compounds were identified: 12 aldehydes, 13 pyrazines, 8 ketones and esters, 9 furans, and 2 acids. 

From the data reported, a general reduction in the concentration of the aforementioned aldehydes in GF breadsticks added with OCP (BS1, BS2, and BS3) could be observed when compared to BSC (control sample without OCP). This could be related to the higher antioxidant activity and phenolic compounds (Figure 1) able to inactivate free radicals, stabilising them through their transfer of a hydrogen atom or a single electron [89]. Therefore, a delay in the oxidation process of the products can be assumed, as was also observed by Difonzo et al. [53] by adding olive leaf extract (OLE) in baked snacks. Even Rutkowska et al. [90] found a reduction in the hydroperoxide content in muffins fortified with increasing concentrations of chokeberry polyphenol extract (ChPE) (0.25, 0.50, and 0.70%) after eight weeks of storage. However, Rutkowska et al. [90] highlighted that there was no direct correlation between the amount of ChPE added and lipid oxidation inhibition. Furthermore, Maillard reactions, as already mentioned, contribute in a significant way to the aroma and flavour of baked foods through the production of a wide range of volatile compounds [69]. 

Strecker aldehydes, furans, pyrazines, and ketones are the main classes of volatile compounds that develop during Maillard reactions, which contribute to conferring the typical aroma to bakery products such as bread, cookies, and cakes [91,92]. Strecker degradation is one of the main stages of Maillard reactions, which occurs between α-di-carbonyl compounds produced by carbohydrate dehydration, or fragmentation, and amino acids, and leads to the production of aldehydes with the corresponding structure of the initial amino acids [92,93]. Notably, 3-methylbutyraldehydes, 2-methylbutanal, and 2-methylpropanal, derive, respectively, from leucine, isoleucine, and valine [94,95], and their concentration was reduced by 75, 65.5, and 59 % in BS3 when compared with the control sample, BSC. 

An opposite trend was found for benzaldehyde, whose concentration increased proportionally to OCP addition, reaching a value of 112.30 µg g^−1^ in BS3 when compared to BSC (5.83 µg g^−1^). Benzaldehyde is another characteristic compound of Maillard reactions [72,96], but from a literature analysis it emerged that this compound is also characteristic of drupaceous fruits; therefore olives and its derived products would justify the increase in its concentration with increasing quantities of OCP added in the GF breadstick formulation [97,98,99,100]. 

Furthermore, furans and pyrazines followed the same trend of Strecker aldehydes, with, generally, a significant decrease in their concentration in enriched GF breadsticks. Among furans, 2-furanmethanol was the most abundant, which gives aromas of toasted caramel and nuts [87]. Pyrazines are usually formed by interaction between the products of Maillard reactions and Strecker degradation and, together with furans, significantly contribute to the flavour of baked products [87,96]. Nevertheless, several studies highlighted the influence of phenolic compounds on the development of volatile compounds in the end products [93,101,102,103,104]. The inhibitory action of polyphenols against aromatic compounds can be regulated by three mechanisms: entrapment of di-carbonyl groups from sugar fragmentation, reaction of their degradation products with aromatic compounds, or they can act as radical scavengers of free radicals involved in Maillard reactions, inhibiting flavour formation [105]. According to Hidalgo et al. [106], the ability of phenols to trap Strecker aldehydes is related to the high nucleophilicity of their atoms. However, their trapping ability is not the same in all aldehydes and the formation of carbonyl–phenol is connected with the structure of both the phenols and aldehydes involved [106]. 

Moreover, Mildner-Szkudlarz et al. [102,107] assessed the inhibitory action of phenolic compounds (mainly gallic and caffeic acids, followed by ferulic acid, catechins, and quercetin) against pyrazine and furan production during bread baking, confirming our results. Similarly, a significant reduction in furans, Strecker aldehydes, and pyrazines occurred in a coffee-canned model system through the addition of di-carbonyl trapping agent, water, and fat-soluble antioxidants and reducing agents (glutathione and sodium sulphite) [103]. 

### 3.5. Sensory Profile

In general, the addition of OCP significantly influenced sensory parameters. The results (Table 5) showed a significant increase in colour intensity proportionally to OCP added in GF breadstick formulation, with the highest values in BS2 (7.50 ± 0.55) and BS3 (6.57 ± 1.25). The result was in line with colorimetric analysis, where a reduction in luminosity (L*) and yellow index (b*) was observed. The same results were found by Cecchi et al. [108] after the addition of olive cake for granola bar manufacturing. No differences were observed for surface and colour uniformity between enriched samples and control, with the exception of sample BS1, due to the manual manufacturing. Regarding flavour, panellists perceived a higher olive hint in BS2 and BS3 than in BS1. Furthermore, BS3 was perceived as less hard and more brittle, confirming the instrumental results on texture profile. With respect to taste, panellists perceived a higher intensity of bitterness and astringency in the BS3 sample, which may be reasonably associated with the highest phenol concentration known to provide bitter and astringent sensations [109,110,111]. Moreover, oiliness perception was greater in the BS2 and BS3 samples, but no differences were perceived among the samples for saltiness. According to the overall pleasantness results, OCP improved the acceptability of GF breadsticks; indeed, BS2 and BS3 received an overall pleasing score, higher than for BS1 and BSC.

## 4. Conclusions

The obtained results demonstrated how OCP addition into GF breadstick formulations was able to enhance the overall quality of the end products through the exploitation of a by-product in a sustainable perspective. The replacement of maize flour with OCP determined an increase in lipids, ash, moisture, and total dietary fibre in the enriched samples. BS2 and BS3 reached a total dietary fibre content of more than 3 g 100 g^−1^, making them suitable to be labelled as a “source of fibre”. The addition of OCP allowed us to improve total phenol content and antioxidant activity, which are supposed to reduce the concentration of some aldehydes, markers of lipids oxidation, and the main volatile classes of Maillard reactions in fortified breadsticks. Finally, OCP addition induced a reduction in hardness and an improvement in overall pleasantness, as evaluated by the sensory evaluation.

## Figures and Tables

**Table 1:** Mean values and standard deviation of the proximate composition and bioactive profile of olive cake powder.

Parameters	OCP
Moisture (g 100 g^−1^)	2.32 ± 0.04
Lipids (g 100 g^−1^)	22.78 ± 0.56
Total dietary fibre (g 100 g^−1^)	23.45 ± 0.18
Proteins (g 100 g^−1^)	7.68 ± 0.12
Ash (g 100 g^−1^)	8.97 ± 0.34
Carbohydrates (g 100 g^−1^)	34.80 ± 0.25
**Bioactive compounds**	
Total phenols (mg GAE g^−1^)	78.23 ± 0.54
DPPH (μmol TE g^−1^)	285.24 ± 11.21
ABTS (μmol TE g^−1^)	346.12 ± 8.11
Total tocopherols (mg kg^−1^)	412.24 ± 0.12

**Table 2:** Mean values, standard deviation, and results of statistical analysis of the proximate composition of GF breadsticks.

Parameters	BSC	BS1	BS2	BS3
Moisture (g 100 g^−1^)	2.90 ± 0.02c	5.02 ± 0.08a	4.18 ± 0.01b	4.98 ± 0.11a
Lipids (g 100 g^−1^)	14.38 ± 0.03b	14.78 ± 0.23b	16.14 ± 0.16a	16.08 ± 0.17a
Total dietary fibre (g 100 g^−1^)	2.72 ± 0.03c	2.90 ± 0.08c	3.16 ± 0.08b	3.59 ± 0.13a
Proteins (g 100 g^−1^)	6.23 ± 0.20a	5.58 ± 0.01b	5.69 ± 0.11b	5.57 ± 0.02b
Ash (g 100 g^−1^)	1.83 ± 0.03c	2.43 ± 0.04b	2.49 ± 0.02b	2.67 ± 0.04a
Carbohydrates (g 100 g^−1^)	71.94 ± 0.20a	69.29 ± 0.21b	68.34 ± 0.25c	67.11 ± 0.33d

Different letters in the same row show significantly different means of data (*p* < 0.05) according to one-way ANOVA and the Tukey’s HSD test. Abbreviations: BSC, control breadsticks without olive cake powder; BS1, BS2, BS3, breadsticks with 1, 2, and 3% olive cake powder replacing maize flour, respectively.

**Table 3:** Mean values, standard deviation, and results of statistical analysis of the colorimetric and textural properties of GF breadsticks.

Parameters	BSC	BS1	BS2	BS3
Colorimetric indexes				
L*	61.60 ± 0.13a	53.31 ± 0.38b	47.18 ± 0.24c	46.07 ± 0.20d
b*	31.89 ± 0.10a	27.22 ± 0.23b	27.33 ± 0.54b	26.05 ± 0.31c
a*	7.83 ± 0.10b	7.15 ± 0.17c	8.49 ± 0.22a	7.99 ± 0.02b
ΔE	-	9.55 ± 0.53a	15.15 ± 0.19b	16.60 ± 0.22c
Textural indexes				
Hardness (N)	19.12 ± 0.72a	19.40 ± 0.32a	18.91 ± 0.75a	12.68 ± 0.16b
Brittleness (mm)	0.08 ± 0.02a	0.09 ± 0.01a	0.10 ± 0.02a	0.07 ± 0.01a

Different letters in the same row show significantly different means of data (*p* < 0.05) according to one-way ANOVA and the Tukey’s HSD test. Abbreviations: BSC, control breadsticks without olive cake powder; BS1, BS2, BS3, breadsticks with 1, 2, and 3% olive cake powder replacing maize flour, respectively.

**Table 4:** Mean values, standard deviation, and results of statistical analysis of the volatile compounds (expressed as μg g^−1^) into GF breadsticks.

Volatile Compounds	BSC	BS1	BS2	BS3
Aldehydes				
2-Methylpropanal	13.04 ± 0.43a	4.89 ± 0.63c	6.85 ± 0.29b	5.35 ± 0.25c
2-Methylbutanal	29.34 ± 1.26a	10.80 ± 0.72c	13.21 ± 0.54b	10.13 ± 0.20c
3-Methylbutyraldehyde	95.50 ± 5.53a	35.33 ± 1.39b	38.24 ± 0.38b	22.95 ± 1.62c
Pentanal	3.74 ± 0.55a	2.10 ± 0.11b	2.33 ± 0.17b	1.96 ± 0.05b
2-Butenal	1.05 ± 0.30a	0.36 ± 0.13b	0.58 ± 0.08b	0.42 ± 0.11b
Hexanal	24.66 ± 2.83a	12.48 ± 0.69b	12.54 ± 0.67b	14.19 ± 0.91b
Heptanal	3.60 ± 0.19a	1.24 ± 0.21c	2.85 ± 0.17ab	1.99 ± 0.70bc
Octanal	1.56 ± 0.46a	0.82 ± 0.19b	1.77 ± 0.13a	1.53 ± 0.09a
*trans*-2-heptenal	10.97 ± 0.12a	9.21 ± 0.18b	9.04 ± 0.77b	7.52 ± 0.38c
Nonanal	0.98 ± 0.12a	0.72 ± 0.07a	0.83 ± 0.20a	0.85 ± 0.20a
*trans*-2-octenal	0.74 ± 0.10a	0.58 ± 0.23a	0.65 ± 0.07a	0.74 ± 0.17a
Benzaldehyde	5.83 ± 0.66d	29.40 ± 1.38c	75.94 ± 4.10b	112.30 ± 11.48a
Ketones and esters				
2-Butanone	10.08 ± 0.92a	1.49 ± 0.0.8b	2.28 ± 0.25b	1.97 ± 0.05b
2-Pentanone	0.62 ± 0.03a	0.17 ± 0.09b	0.15 ± 0.03b	0.14 ± 0.02b
2,3-Butanedione	15.30 ± 0.86a	7.12 ± 0.38c	7.39 ± 0.29c	11.00 ± 0.39b
2,3-Pentanedione	12.95 ± 0.83a	5.24 ± 0.62c	7.49 ± 0.49b	8.18 ± 0.58b
2-Heptanone	6.76 ± 0.90a	0.54 ± 0.07c	1.99 ± 0.22b	1.22 ± 0.26bc
2-Octanone	0.35 ± 0.02a	0.15 ± 0.03b	0.17 ± 0.03b	0.10 ± 0.02b
1-Octen-3-one	0.79 ± 0.30bc	0.77 ± 0.14c	1.24 ± 0.06ab	1.29 ± 0.10a
Ethanone, 1-(2-furanyl)	1.01 ± 0.15a	0.44 ± 0.06b	0.47 ± 0.05b	0.58 ± 0.03b
Acids				
Propanoic acid	1.11 ± 0.06a	1.15 ± 0.10b	1.68 ± 0.07b	1.05 ± 0.06b
Acetic acid	0.30 ± 0.16b	0.14 ± 0.04b	0.26 ± 0.03b	0.83 ± 0.12a
Furans				
Furan	4.25 ± 0.85a	1.07 ± 0.33b	2.22 ± 0.16b	2.25 ± 0.12b
Furan, 2-methyl-	18.69 ± 1.17a	2.44 ± 0.25c	1.99 ± 0.20c	19.01 ± 0.64b
Furan, 2-ethyl-	1.56 ± 0.13a	0.16 ± 0.04c	0.41 ± 0.07b	0.56 ± 0.05b
Furan, 2-pentyl-	1.55 ± 0.17a	0.73 ± 0.03b	1.41 ± 0.09a	0.74 ± 0.04b
2(3H)-Furanone, 5-methyl	0.46 ± 0.03a	0.16 ± 0.05b	0.13 ± 0.03b	0.19 ± 0.02b
2-Furancarboxaldehyde	11.36 ± 0.79a	4.63 ± 0.51c	5.21 ± 0.57c	6.89 ± 0.48b
2-Furancarboxaldehyde,5-methyl	0.69 ± 0.07b	0.73 ± 0.08b	1.05 ± 0.18a	0.40 ± 0.04c
2(3H)-Furanone, dihydro-	1.27 ± 0.14a	0.73 ± 0.04b	0.62 ± 0.04b	0.89 ± 0.18b
2-Furanmethanol	40.50 ± 0.79a	10.49 ± 0.54bc	9.21 ± 0.55c	11.84 ± 0.77b
Sulfurs				
Carbon disulfide	1.41 ± 0.17a	1.03 ± 0.05a	1.29 ± 0.13a	1.44 ± 0.36a
Pirazines				
Pyrazine	7.82 ± 0.27a	1.97 ± 0.54b	1.33 ± 0.08b	1.75 ± 0.46b
Pyrazine, methyl-	31.46 ± 0.86a	12.26 ± 1.35b	12.32 ± 0.52b	6.92 ± 1.87c
Pyrazine, 2,6-dimethyl-	6.02 ± 0.30a	3.23 ± 0.13b	3.45 ± 0.14b	2.48 ± 0.26c
Pyrazine, ethyl-	14.09 ± 0.62a	6.41 ± 0.56b	5.46 ± 0.21b	3.25 ± 0.04c
Pyrazine, 2,3-dimethyl-	5.56 ± 0.42a	2.58 ± 0.47b	1.71 ± 0.13c	0.77 ± 0.03d
Pyrazine, 2-ethyl-6-methyl-	3.53 ± 0.18a	1.44 ± 0.20b	1.78 ± 0.44b	1.81 ± 0.12b
Pyrazine, 2-ethyl-5-methyl-	7.28 ± 0.26a	4.95 ± 0.78b	4.83 ± 0.48b	4.92 ± 0.36b
Pyrazine, 2-ethyl-3-methyl-	4.66 ± 0.26a	2.29 ± 0.03b	1.80 ± 0.06c	1.11 ± 0.04d
Pyrazine, propyl-	0.74 ± 0.08a	0.25 ± 0.02b	0.22 ± 0.03b	0.23 ± 0.04b
Pyrazine, ethenyl	0.74 ± 0.10a	0.58 ± 0.23b	0.65 ± 0.07b	0.74 ± 0.17b
Pyrazine, 3-ethyl-2,5-dimethyl-	1.10 ± 0.09a	0.45 ± 0.08b	0.47 ± 0.06b	0.34 ± 0.03b
Pyrazine, 2-ethyl-3,5-dimethyl-	0.77 ± 0.08ab	0.37 ± 0.03a	0.34 ± 0.04ab	0.27 ± 0.05b
Pyrazine, 2-ethenyl-6-methyl	0.84 ± 0.08a	0.62 ± 0,05a	0.74 ± 0.10a	0.84 ± 0.12a

Different letters in the same row show significantly different means of data (*p* < 0.05) according to one-way ANOVA and the Tukey’s HSD test. Abbreviations: BSC, control breadsticks without olive cake powder; BS1, BS2, BS3, breadsticks with 1, 2, and 3% olive cake powder replacing maize flour, respectively.

**Table 5:** Mean values, standard deviation, and results of statistical analysis of the sensorial analysis of GF breadsticks.

Parameters	BSC	BS1	BS2	BS3
Appearance				
Colour uniformity	7.83 ± 0.82a	5.67 ± 0.82b	7.08 ± 0.67a	8.25 ± 0.61a
Colour intensity	1.92 ± 0.20c	4.92 ± 0.20b	7.50 ± 0.55a	7.92 ± 0.80a
Surface uniformity	7.17 ± 0.98ab	5.83 ± 0.98b	7.67 ± 0.82a	6.67 ± 1.37ab
Flavour				
Olive	-	1.67 ± 1.21b	5.33 ± 1.03a	6.42 ± 0.92a
Texture				
Hardness	6.42 ± 1.20a	5.50 ± 1.38a	5.50 ± 1.23a	3.58 ± 0.80b
Brittleness	4.00 ± 0.63b	4.17 ± 0.98b	6.50 ± 0.55a	7.00 ± 0.84a
Taste				
Salty	1.91 ± 0.92a	2.42 ± 0.67a	2.33 ± 0.52a	2.50 ± 0.55a
Bitterness	0.58 ± 0.49c	2.25 ± 0.88b	3.42 ± 1.02b	5.50 ± 1.05a
Astringency	0.33 ± 0.52b	1.00 ± 1.27b	0.83 ± 0.98b	3.67 ± 1.97a
Oiliness	1.08 ± 1.02b	0.92 ± 0.67b	1.42 ± 1.11ab	2.67 ± 1.03a
Overall pleasantness	5.42 ± 0.92b	5.92 ± 0.49b	7.67 ± 0.75a	7.92 ± 0.19a

Different letters in the same row show significantly different means of data (*p* < 0.05) according to one-way ANOVA and the Tukey’s HSD test. Abbreviations: BSC, control breadsticks without olive cake powder; BS1, BS2, BS3, breadsticks with 1, 2, and 3% olive cake powder replacing maize flour, respectively.

## Data Availability

The data presented in this study are available on request from the corresponding author.
